# Severe Loss of Suitable Climatic Conditions for Marsupial Species in Brazil: Challenges and Opportunities for Conservation

**DOI:** 10.1371/journal.pone.0046257

**Published:** 2012-09-28

**Authors:** Rafael D. Loyola, Priscila Lemes, Frederico V. Faleiro, Joaquim Trindade-Filho, Ricardo B. Machado

**Affiliations:** 1 Department of Ecology, Universidade Federal de Goiás, Goiânia, Goiás, Brazil; 2 Graduate Program in Ecology and Evolution, Universidade Federal de Goiás, Goiânia, Goiás, Brazil; 3 Departament of Zoology, Universidade de Brasília, Brasília, Distrito Federal, Brazil; University of Kent, United Kingdom

## Abstract

A wide range of evidences indicate climate change as one the greatest threats to biodiversity in the 21st century. The impacts of these changes, which may have already resulted in several recent species extinction, are species-specific and produce shifts in species phenology, ecological interactions, and geographical distributions. Here we used cutting-edge methods of species distribution models combining thousands of model projections to generate a complete and comprehensive ensemble of forecasts that shows the likely impacts of climate change in the distribution of all 55 marsupial species that occur in Brazil. Consensus projections forecasted range shifts that culminate with high species richness in the southeast of Brazil, both for the current time and for 2050. Most species had a significant range contraction and lost climate space. Turnover rates were relatively high, but vary across the country. We also mapped sites retaining climatic suitability. They can be found in all Brazilian biomes, especially in the pampas region, in the southern part of the Brazilian Atlantic Forest, in the north of the Cerrado and Caatinga, and in the northwest of the Amazon. Our results provide a general overview on the likely effects of global climate change on the distribution of marsupials in the country as well as in the patterns of species richness and turnover found in regional marsupial assemblages.

## Introduction

As a result of Earth's climate warming and changes in precipitation regimes, the scientific community has a consensual agreement that conservation strategies for managing biodiversity must anticipate the impacts of climate change to be effective [1]. Most studies on climate change have been developed at local scales and use experimental, manipulative schemes, despite the much broader geographical scales at which these changes are expected to affect biodiversity patterns [2]. On the other hand, studies addressing the effects of climate change on biodiversity at continental scales are based on how species' distribution will be potentially driven by such changes, usually inferred through species distribution models [3]. These models are based on different mathematical functions that establish correlations between species' occurrences and environmental variables and, once these correlations were established, make it possible to project the model into future climates to predict species responses (assuming species' niche itself will not respond to these changes) [4].

Species distribution models have been used to predict the current and future species' distributions [5]. However, different methods for modeling species distribution and different climate models (i.e. the coupled Atmosphere-Ocean General Circulation Models, AOGCMs) may produce very distinct results increasing the uncertainties among predictions and their applicability to conservation planning [6], [7]. Consequently, measuring and mapping uncertainties are necessary to increase the quality of conservation plans [8], [9].

Brazil corresponds to half of South America, and concentrates more than 13% of the world's biota – in particular, *ca.* 11% of the world's mammals [10]. The country holds at least 55 marsupial species ranging from small (*ca.* 10 g) to large species (*ca.* 4 kg) distributed mostly in forest areas such as the Amazon and the Atlantic Forest [11]. However, we still know little about the distribution of marsupials in the countryside, especially in the Brazilian Cerrado, and in the Brazilian Pantanal [11]. This lack of knowledge reinforces the importance of generating species distribution models for this group. Further, marsupials are highly threatened by forest fragmentation, although we also still lack detailed information about marsupial responses to this process [12]. Such vulnerability highlights the need for studies about the effects of global changes (e.g. climate and land use changes) on the group to develop strategies for climate change adaptation related to mammal conservation in Brazil.

Here we present a comprehensive overview on the likely effects of climate change on the distribution of marsupial species inhabiting Brazil and on the patterns of marsupial species richness and turnover. We also highlight sites in which the retention of suitable climatic conditions could minimize climate-driven extinction risk for marsupials.

## Materials and Methods

Taxonomy of Brazilian marsupials is still incipient, and some species have been changing their taxonomy given the increasing volume of studies on this mammal order in Brazil and its neighbor countries. Here we followed Rossi *et al.* [13] and Gardner [14].

We downloaded extent of occurrence maps of all the 55 marsupial species that occur in Brazil from the International Union for Conservation of Nature and Natural Resources (IUCN) database (www.iucnredlist.org). We overlapped these maps for each species into an equal-area grid (0.25×0.25 degrees of latitude/longitude) that covered the full extent of the country [15]. Then, we built a species by grid cell matrix, considering presences and absences of species inside grid cells. All 55 species had at least ten occurrences, which reduces model bias.

We obtained current climatic data from the WorldClim database (www.worldclim.org/current) and future climatic scenarios from CIAT (ccafs-climate.org) through WorldClim website. The Intergovernmental Panel on Climate Change (IPCC) 's Fourth Assessment Report (AR4) developed these future scenarios [16]. For each species we modeled distribution as a function of four climatic variables: annual mean temperature, temperature seasonality (standard deviation * 100), annual precipitation, and precipitation seasonality (coefficient of variation). These current climatic data were generated by interpolated climate data from 1950–2000 periods. For future climatic conditions, we used climate variables (year 2050) from four Atmosphere-Ocean General Circulation Models (AOGCMs) of the A2a and B2a green house gases emission scenarios (CCCMA-CGCM2, CSIRO-MK2.0, UKMO-HADCM3, and NIESS99) that were generated by the application of delta downscaling method on the original data from the IPCC Fourth Assessment Report (provided by International Centre for Tropical Agriculture at ccafs-climate.org). We re-scaled both current and future climate variables to our grid resolution.

We used presence and absence derived from species occurrences and climatic variables to model species distributions. We fitted six modeling methods, which differ both conceptually and statistically [4], and applied the ensemble forecasting approach within each set (see text below). We used Generalized Linear Models – GLM [17], Generalized Additive Models – GAM [18], Multivariate Adaptive Regression Splines – MARS [19], Random Forest [20], Artificial Neural Networks – ANN [21], and Generalized Boosting Regression Models – GBM [22].

We partitioned randomly presence and absence data of each species in 75% to calibration (or train) and 25% to validation (or test) and repeated this process 10 times (i.e. a cross-validation) maintaining the observed prevalence of each species. We converted continuous predictions in presence and absences finding the threshold with maximum sensitivity and specificity values in the receiver operating characteristic (or simply ROC curve). After this, we calculated the True Skill Statistics (TSS) to evaluate model performance [23]. The TSS range from −1 to +1, where values equal +1 is a perfect prediction and values equal or less of zero is a prediction no better than random [23].

We did the ensembles of forecasts to produce more robust predictions and reduce the model variability owing to the modeling methods applied and climate models used [7], [9], [24], [25]. We projected distributions into future climate and obtained 240 projections per species within each set of methods (6 modeling methods×4 climate models×10 randomly partitioned data) and 60 projections per species for current climatic conditions (6 modeling methods×10 randomly partitioned data) – this allowed us to generate a frequency of projections in the ensemble. We then generated the frequency of projections weighted by the TSS statistics for each species and timeframe within each set of methods. We considered the presence of a species only in cells with 50% or more of frequency of projections, but we hold a continuous value when this occurred.

Finally, we calculated species turnover between current and future species distributions in each cell as (G+L)/(SR+G), where “G” was the number of species gained, “L” the number of species lost and “SR” is the current species richness found in the cell. Then we used the total sum of squares from a two-way Analysis of Variance (ANOVA) without replication to quantify the uncertainty associated to each cell following the protocol recently proposed by [9]. We did the ANOVA using species richness as the response variable, and modeling methods and climate models as factors. Finally, we calculated the percent of variation found in each cell relative to the total uncertainty found in all cells to generate a measure of model uncertainty.

To evaluate in which sites a strategic investment in research and conservation of marsupials would be more adequate, we calculated the percent of species retaining suitable climatic conditions in each grid cell. This value was obtained by adding the number of species that occur in the cell in the present plus the number of species that were predicted to remain in that cell in the future, divided by the species richness found in the cell in the present [26].

Finally, to assess how much species richness and turnover geographic patterns would change if deforested areas were removed from the analysis both now and in 2050 we developed a spatial model of land conversion, using the Cerrado Biodiversity Hotspot as a case study. We modeled land conversion with variables from different sources. We compared the Cerrado land use between 2002 and 2008 (siscom.ibama.gov.br/monitorabiomas/index.htm) to generate a matrix of transition probability between native areas to anthropic areas. Then, we modeled the land conversion with the module Land Change Modeler - LCM, available in Idrisi Taiga Version [27], using these explanatory variables: digital elevation model and annual accumulated precipitation (data available at www.worldclim.org), proximity to roads, proximity to recent deforested areas and proximity to cities (data available at mapas.mma.gov.br/i3geo/datadownload.htm). LCM is a machine learning procedure that uses Markov Chains to project future land-use conditions. To evaluate model precision, we inverted the maps from 2002 and 2008 and the expected land-use was projected back into 1990. After this, we generated a total of 458 control points to cover the entire Cerrado by doing a visual inspection of MrSID images from 1990 (see zulu.ssc.nasa.gov/mrsid). Finally, we predicted land conversion in 2050 with a spatial resolution close to 500×500 m.

## Results

**Figure 1:**
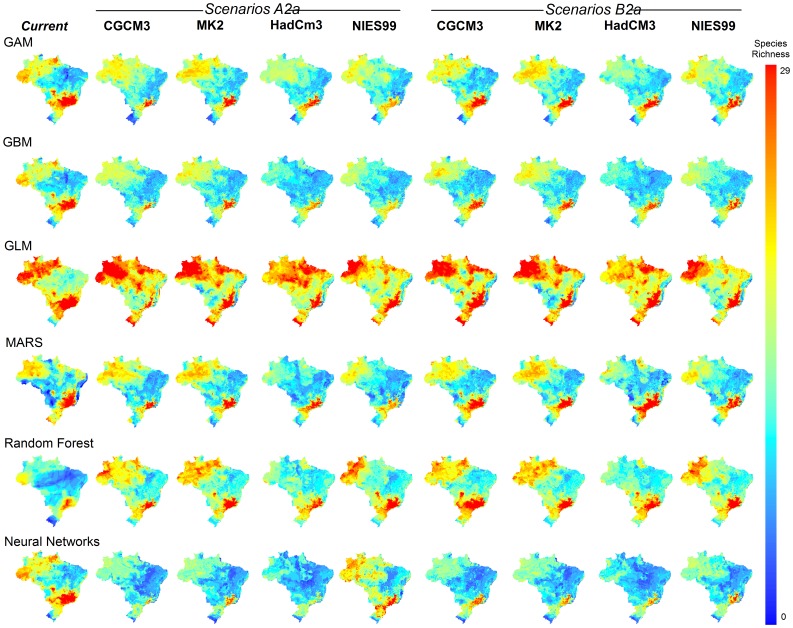
Marsupial species richness patterns in Brazil (current and future, 2050) forecasted by species distribution models generated by different modeling methods (Generalized Additive Models, GAM; Generalized Boosting Regression Models, GBM; Generalized Linear Models, GLM; Multivariate Adaptive Regression Splines, MARS; Artificial Neural Networks, ANN; and Random Forest), climate models (CGCM3, MK2, HadCm3, NIES99), and green house gases emission scenarios (optimist, B2a, and pessimist, A2a). See text for further details.

**Figure 2:**
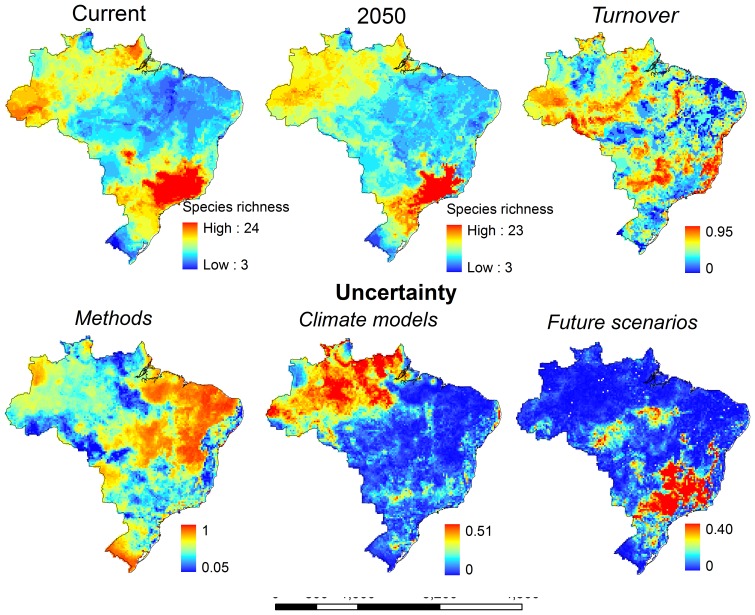
Consensus map of marsupial species richness in Brazil for current and future climatic conditions, mean turnover forecasted by model projections, and geographic patterns of model uncertainty arising from different sources: modeling methods, climate models, and green house gases emission scenarios. See text for further details.

For most species, TSS value was relatively high (TSS ± SD = 0.77±0.14), indicating good model fit. Patterns of marsupial species richness varied depending on the methods employed to model species distributions and the climate models used to project future climatic conditions (Fig. 1). Modeling methods accounted for 62.6% of the variation among projections, whereas climate models explained 10.3% of such variation. Greenhouse gases emission scenarios contributed little to variation among projections (2.7%). Uncertainty arising from modeling methods was high in the northeast, south and in central Brazil (regions with lower species richness). Uncertainties linked to climate models were higher in the north (Fig. 2).

For current time, all models indicated high species richness in the southeast of Brazil, and low richness in the south and northeast of the country, despite variation among projections (Fig. 1). GLM were an exception, indicating high richness in the northern region of Brazil. As for current time, all models forecasted species' range contraction, regardless the emission scenario (Fig. 1). Our consensual model projections (Fig. 2) forecasted species range shifts that culminate also with high richness in the southeast both for current time and for 2050. Species' range contraction was high (67% of contraction on average), although our models did not forecast species extinction until 2050 (Table 1). Although we did not observe a dramatic change in the pattern of species richness, turnover was high across the country, varying from 0% up to 95% of change in species composition. The western portion of the Brazilian Amazon, central Brazil, and the Brazilian Atlantic Forest should expect high species turnover (Table 1, Fig. 2).

**Table 1:** Mean species richness of marsupials in Brazil (S) projected for current and future climatic conditions, different between future & current species richness (Δ), mean turnover, and percent variation (median) of species range size and its interquartile deviation obtained in each Green house gases emission scenario, modeling method, and climate model.

Emission scenario	Modeling method	Climate Model	Species richness (current climate)	Species richness (future climate)	Δ species richness	Turnover	% Range size variation (interquartile deviation)
A2a	GAM	CCCMA-CGCM2	11.28	10.79	0.49	0.50	−44.35 (51.98)
		CSIRO-MK2	11.28	10.63	0.65	0.37	−26.75 (30.59)
		HCCPR-HadCM3	11.28	9.88	1.40	0.55	−52.76 (40.44)
		NIES99	11.28	10.01	1.27	0.48	−30.77 (41.22)
	GBM	CCCMA-CGCM2	10.85	9.46	1.39	0.45	−44.97 (44.41)
		CSIRO-MK2	10.85	9.85	1	0.38	−26.93 (30.14)
		HCCPR-HadCM3	10.85	8.27	2.57	0.54	−49.66 (28.88)
		NIES99	10.85	8.97	1.87	0.48	−39.03 (30.43)
	GLM	CCCMA-CGCM2	14.2	15.4	−1.2	0.49	−33.19 (41.91)
		CSIRO-MK2	14.2	14.78	−0.58	0.35	−7.76 (26.26)
		HCCPR-HadCM3	14.2	15.05	−0.85	0.53	−34.12 (60.03)
		NIES99	14.2	14.21	−0.01	0.49	−21.18 (42.1)
	MARS	CCCMA-CGCM2	11.65	10.34	1.31	0.52	−52.04 (41.18)
		CSIRO-MK2	11.65	10.83	0.82	0.40	−28.72 (26.03)
		HCCPR-HadCM3	11.65	8.86	2.79	0.57	−46.32 (29.14)
		NIES99	11.65	9.21	2.44	0.50	−34.75 (40.05)
	RF	CCCMA-CGCM2	8.53	8.22	0.31	0.43	−21.17 (38.52)
		CSIRO-MK2	8.53	8.36	0.16	0.36	−7.83 (33.13)
		HCCPR-HadCM3	8.53	7.34	1.19	0.54	−35.58 (49.38)
		NIES99	8.53	8.27	0.25	0.45	−0.21 (0.39)
	ANN	CCCMA-CGCM2	14.42	11.83	2.59	0.45	−21.17 (38.52)
		CSIRO-MK2	14.42	12.15	2.27	0.37	−7.83 (33.13)
		HCCPR-HadCM3	14.42	10.16	4.26	0.51	−35.58 (49.38)
		NIES99	14.42	11.66	2.76	0.47	−21.04 (38.79)
B2a	GAM	CCCMA-CGCM2	11.28	10.77	0.51	0.32	−4.21(23.94)
		CSIRO-MK2	11.28	10.86	0.42	0.35	−20.24 (30.20)
		HCCPR-HadCM3	11.28	9.64	1.64	0.52	−38.78 (39.34)
		NIES99	11.28	10.53	0.75	0.42	−30.70 (32.26)
	GBM	CCCMA-CGCM2	10.85	10	0.85	0.38	−22.36 (23.24)
		CSIRO-MK2	10.85	9.97	0.88	0.37	−29.68 (27.66)
		HCCPR-HadCM3	10.85	8.47	2.37	0.53	−42.72 (35.93)
		NIES99	10.85	9.38	1.47	0.45	−34.01 (26.55)
	GLM	CCCMA-CGCM2	14.2	15.05	-0.85	0.31	1.49 (19.73)
		CSIRO-MK2	14.2	14.44	-0.24	0.35	−13.91 (26.46)
		HCCPR-HadCM3	14.2	14.85	-0.65	0.52	−25.3 (56.3)
		NIES99	14.2	13.59	0.61	0.44	−22.71 (34.9)
	MARS	CCCMA-CGCM2	11.65	10.98	0.67	0.37	−10.82 (20.23)
		CSIRO-MK2	11.65	10.97	0.68	0.38	−21.31 (23.62)
		HCCPR-HadCM3	11.65	8.9	2.76	0.55	−36.47 (27.53)
		NIES99	11.65	10	1.65	0.45	−32.35 (25.84)
	RF	CCCMA-CGCM2	8.53	8.49	0.04	0.38	−0.03 (0.23)
		CSIRO-MK2	8.53	8.42	0.11	0.37	−10.63 (31.19)
		HCCPR-HadCM3	8.53	7.49	1.04	0.54	−34.87 (41.99)
		NIES99	8.53	8.63	−0.1	0.42	−12.87 (38.57)
	ANN	CCCMA-CGCM2	14.42	12.60	1.82	0.39	−3.34 (23.3)
		CSIRO-MK2	14.42	12.12	2.30	0.37	−10.63 (31.19)
		HCCPR-HadCM3	14.42	10.62	3.80	0.52	−34.87 (41.99)
		NIES99	14.42	11.99	2.43	0.45	−12.77 (38.57)

Generalized Additive Models, GAM; Generalized Boosting Regression Models, GBM; Generalized Linear Models, GLM; Multivariate Adaptive Regression Splines, MARS; Artificial Neural Networks, ANN; and Random Forest, RF.

**Figure 3:**
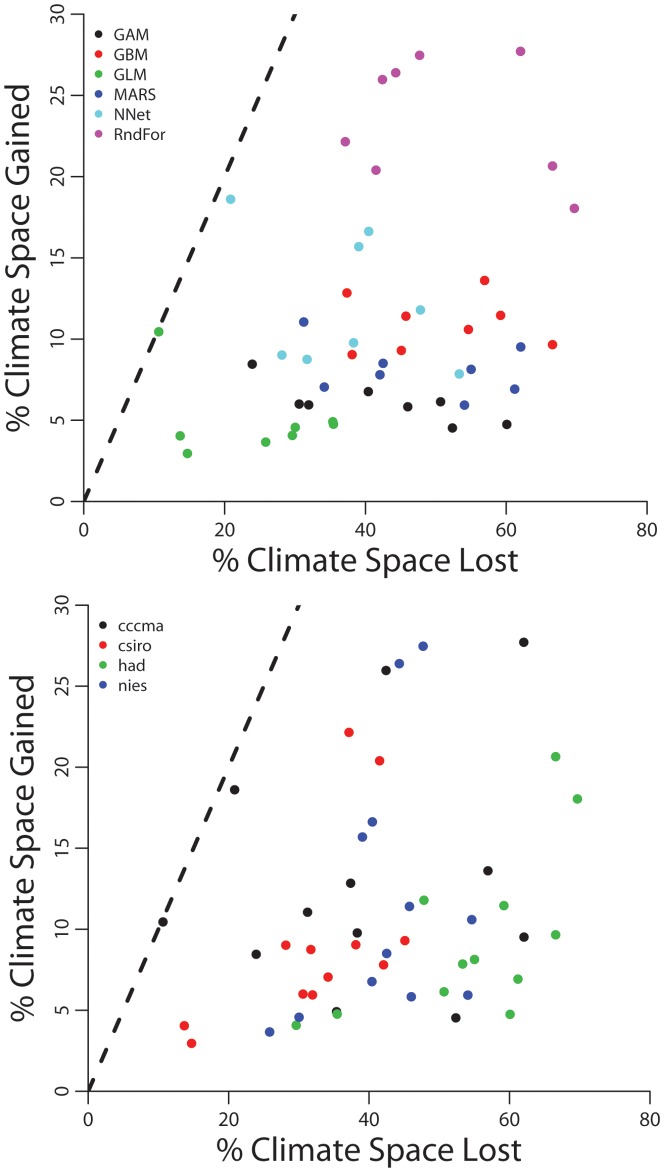
Proportion of climatically suitable sites (grid cells) that may be lost or gained by marsupial species in Brazil, according to six modeling methods (Generalized Additive Models, GAM; Generalized Boosting Regression Models, GBM; Generalized Linear Models, GLM; Multivariate Adaptive Regression Splines, MARS; Artificial Neural Networks, ANN; and Random Forest) and four climate models (CGCM3, MK2, HadCm3, NIES99). Values are median percentages of grid cells lost or gained for all marsupial species in a consensus of green house gases emissions scenarios (B2a and A2a). The line indicates what is expected if gains or losses of climatically suitable sites were proportional and happen by chance.

Marsupials should loose much more climatic space (sites with suitable climate) than gain it, and this result is consistent even under different combinations of modeling methods and climate models (Table 1, Fig. 3). Nevertheless, there was variation in the magnitude of the loss/gain of climate space among modeling methods and climate models (Fig. 3). Random forest projected higher gains in climate space, whereas other methods showed similar projections. Similarly, climate model generated by the Hadley Centre UK (HadCm3) projected the higher losses of climate space (Fig. 3).

**Figure 4:**
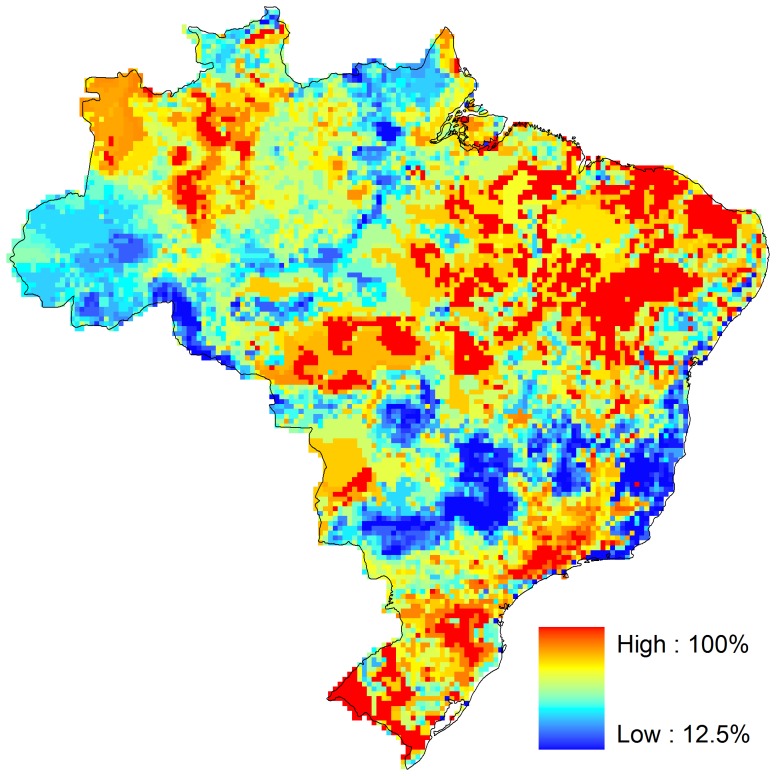
Percentage of species predicted to retain climatic suitability under a consensus of modeling methods, climate models, and green house gases emissions scenarios. Values are median percentages of species in each grid cell as forecasted by a consensus of all modeling methods (Generalized Additive Models, GAM; Generalized Boosting Regression Models, GBM; Generalized Linear Models, GLM; Multivariate Adaptive Regression Splines, MARS; Artificial Neural Networks, ANN; and Random Forest), climate models (CGCM3, MK2, HadCm3, NIES99), and green house gases emissions scenarios (B2a and A2a). See text for further details.

Regions with the highest retention of suitable climate space in Brazil overlap with those regions with low species turnover (compare Figs. 3 & 4). All Brazilian biomes had regions with high retention of adequate climate space. These regions are located in the southern part of the Atlantic Forest, southeast Pantanal, northern Cerrado and Caatinga, and eastern Amazon (Fig. 4).

**Figure 5:**
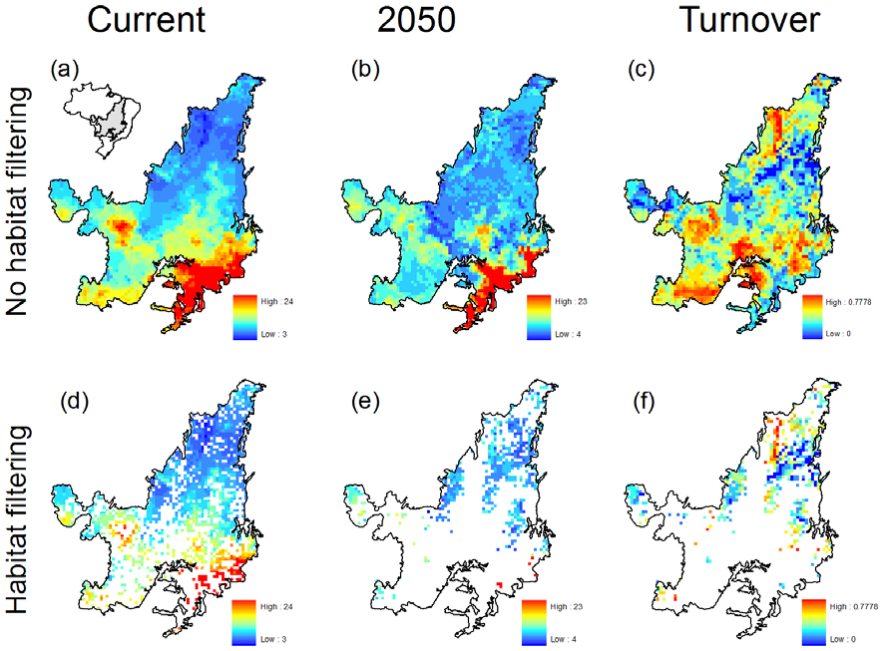
Consensus map of marsupial species richness in the Cerrado Biodiversity Hotspot for current and future climatic conditions, and mean turnover forecasted by model projections. Maps show patterns expected for the whole domain, no habitat filtering (a–c), and with habitat filtering (d–e). Habitat filtering followed the predictions of a spatial model of land conversion for the Cerrado, developed in this study. See text for further details.

Figure 5 shows the effect of habitat filtering (i.e. of including current and future land conversion) in our model predictions in the Cerrado region. Whereas the pattern in species richness and turnover remains essentially the same, the absolute number of species found in a given cell tends to reduce as a consequence of habitat loss (Fig. 5c–e). This is clearly depicted from Fig. 5d, in which future land conversion greatly reduces the amount of available habitats in the region.

## Discussion

We showed that marsupials in Brazil might loose considerable climatically suitable area within their geographic range, loosing climate space towards the year 2050. This projection holds even under different combinations of modeling methods, climate models, and green house gases emission scenarios used to generate species distribution models. Our results have important implication to mammal conservation, and for the conservation of marsupial species, in particular.

First, the use of ensemble of forecasts is preferred as oppose to model species distribution based on only one modeling method (e.g. MaxEnt) and climate model. This is because there is high variation (uncertainty) around model projections, as show here for marsupials, and elsewhere for other taxonomic groups [9], [25], [26], [28]. Ensembles of model projections keep only the consensus-projected areas, minimizing variation among models [7]. This is especially important for conservation purposes given that model uncertainty may mislead conservation efforts ending up being cost-ineffective.

Second, conservation actions based on our marsupial species distribution models must be taken with prudence especially in the central and northeast Brazil as well as in the Amazon, because model uncertainty is higher in these regions. Uncertainties arising from distinct green house gases emission scenarios may be neglected because their level is fairly low, as suggested in other papers [9]. Nevertheless, most Brazilian biomes might hold climatically suitable sites in which conservation action would succeed. Implementing new protected areas in these sites are highly recommended instead of implementing them in climatically unstable sites. Regions with high climate anomalies could be tracked to indicate where we should focus our attention for species extinction risk [29], but should be avoided in decision making processes as there are no guarantee on the maintenance of viable populations there. Policy maker should therefore focus on sites retaining suitable climate [26].

For marsupial species, in particular, attention should be directed to the Cerrado (central Brazil), Pantanal (southwest), Atlantic Forest (east) and the Pampas (south). These areas hold considerable extensions of suitable climates combined with low model uncertainty. We are not saying the Amazon, for instance, is not important, but action in this region require more extensive studies, based on solid field samples, habitat models and landscape assessment. Sites with high turnover rates could be also important targets for conservation action. However, protecting these sites would imply in allocating conservation resources to a constantly changing community structure, precluding an objective conservation goal such as safeguarding viable populations. In this case, habitat and population monitoring would work better, especially to indicate future habitat corridors or stepping-stones for building a regional conservation strategy. Lastly, high turnover rates might imply in unstable provisioning of ecosystem services provided by marsupial such as nutrient cycling and seed dispersal. Managers should take this into consideration when developing action plans for the group.

Even more important is to consider land conversion when planning for on-the-ground conservation actions. Our example with the Cerrado showed that land use changes might reduce dramatically the amount of available habitats for marsupial species. Therefore, under such scenarios of land conversion, loss of climatically suitable areas will be even higher than our initial projections. This would also have a significant effect on resource allocation for marsupial conservation. Ultimately, land use changes are a pivotal source of uncertainty driving species distribution towards worse scenarios in the future. Here, we forecasted high species richness in southeast Brazil, which is the region with greater economic development and human population density in the country. Future analysis on the effects of species distribution under climate change should also include land use change, especially in regions like these, as a major source of uncertainty in the modeling framework. As for our projections, the southeast of Brazil, although climatically adequate, may have already lost some of marsupial species (or at least viable or large populations of these species) because of the more intense human occupation (see also Diniz-Filho *et al.* [30]).

As in any study attempting to model species distributions, this one has its own caveats. First our models assume marsupial species are in equilibrium with current climate and have unlimited dispersal to tackle suitable climates as they move in the geographic space. These are simple assumptions allowing us to model all species distribution at a time. But marsupial dispersal is clearly limited by the composition of the matrix in a fragmented context found in most regions of Brazil [31], [32]. Second, we predicted future species distribution assuming that the vegetation types in Brazil will remain in the same regions of the current distribution, but such changes can occur in South America [33]. This assumption can affect our species distribution model predictions. Species' range shift outside of the current limitation of Brazil or their preferential habitat cannot be measure by our methods. Yet, we believe that this assumption will have little effect in our predictions because these changes would be a real problem only for narrow ranged species that are habitat specialists which is not the case of Brazilian marsupials, in general [11], but see [34]. Third, land use changes were not fully integrated in the analyses – except for the Cerrado region. As discussed above, the dynamic nature of land conversion is a key factor driving species' distribution in space and time. Lastly, in our study we implicitly assume that the marsupial fauna is Brazil is relatively well known. However, it is likely that many other unknown species remain [35]. For mammals, in particular, current estimates suggest an increase of up to 6% in species description, most being small-ranged species [35]. While creative solutions could be applied to decide where to allocate conservation efforts in the face of such lack of knowledge [36], uncertainty about the existence of species, the so-called Linnaean shortfall, remains as a difficult-to-tackle problem in species distribution modeling and conservation assessment.

To sum up, our results provide a general overview on the likely effects of global climate change on the distribution of marsupials in Brazil as well as in the patterns of species richness and turnover found in regional marsupial assemblages. Forecasts are not good - especially if future projections of land conversion are integrated to our results - but we do have time and building capacity to think hard about the problem and find solution for climate change adaptation concerning the fauna of a megadiverse country.
